# STN-DBS does not increase the risk of sialorrhea in patients with advanced Parkinson’s disease

**DOI:** 10.1038/s41531-022-00348-1

**Published:** 2022-06-29

**Authors:** Francesco Bove, Danilo Genovese, Martina Petracca, Tommaso Tufo, Danila Pisani, Maria Rita Lo Monaco, Anna Rita Bentivoglio, Paolo Calabresi, Carla Piano

**Affiliations:** 1 Neurology Unit, Fondazione Policlinico Universitario A. Gemelli IRCCS, Rome, Italy; 2 Department of Neuroscience, Università Cattolica del Sacro Cuore, Rome, Italy; 3 Neurosurgery Unit, Fondazione Policlinico Universitario A. Gemelli IRCCS, Rome, Italy; 4 Medicine of Ageing, Fondazione Policlinico Universitario A. Gemelli IRCCS, Rome, Italy; 5 Department of Geriatrics, Università Cattolica del Sacro Cuore, Rome, Italy

**Keywords:** Parkinson's disease, Neurological disorders, Neurology

## Abstract

The aims of this study were to assess the incidence rate and risk factors for sialorrhea in the long-term follow-up in a cohort of 132 patients with advanced Parkinson’s disease [88 with deep brain stimulation (DBS) and 44 on medical treatment]. The incidence rate of sialorrhea did not differ between the two groups; male sex, Hoehn and Yahr stage and dysphagia resulted risk factors for sialorrhea. These findings indicate that DBS does not increase the risk of developing sialorrhea.

In patients with advanced Parkinson’s disease (PD), deep brain stimulation of the subthalamic nucleus (STN-DBS) is a well-recognized effective treatment on motor symptoms in both short- and long-term follow-up^1,2^. Bilateral STN-DBS also improves a variety of non-motor symptoms^3^, although is not well defined the effect on autonomic and gastrointestinal symptoms^4^. Among these, sialorrhea is a frequent and disabling symptom in patients with PD^5,6^, with a profound effect on the quality of life^7,8^. No study has been specifically designed to test the effects of DBS on sialorrhea development, and conflicting results about the influence of DBS on swallowing function are reported^9,10^. Indeed, increased drooling and dysphagia were reported as adverse effects in different cohorts of stimulated patients^11,12^, but it is unclear if these are only consequences of advanced PD rather than stimulation-induced side effects. The objective of the present study was to evaluate the effect of STN-DBS on the development of sialorrhea in PD patients, assessing the incidence rate and risk factors for sialorrhea in the long-term follow-up.

A total of 248 patients with advanced PD were retrospectively retrieved from our databases, and 132 (88 with bilateral STN-DBS and 44 on medical treatment) met the inclusion and exclusion criteria of the study (Fig. 1a). In Supplementary Table 1 are reported baseline demographic and clinical data of the included patients. Apart from the axial subscore of the UPDRS-III in on-medication condition, slightly higher for the DBS group, and therapy with antidepressants, more frequent among stimulated patients, the two groups were well balanced at baseline. The prevalence of sialorrhea at baseline did not differ significantly between the two groups: 19.3% in the DBS group and 11.4% in the control group (*p* = 0.2). The mean observational period after the baseline was higher for the DBS group, compared to the control group (7.9 ± 6.2 years vs. 4.6 ± 2.9 years respectively, *p* = 0.02). The longest follow-up period reached 24 years for DBS patients and 17 years for medically managed patients.

**Fig. 1:**
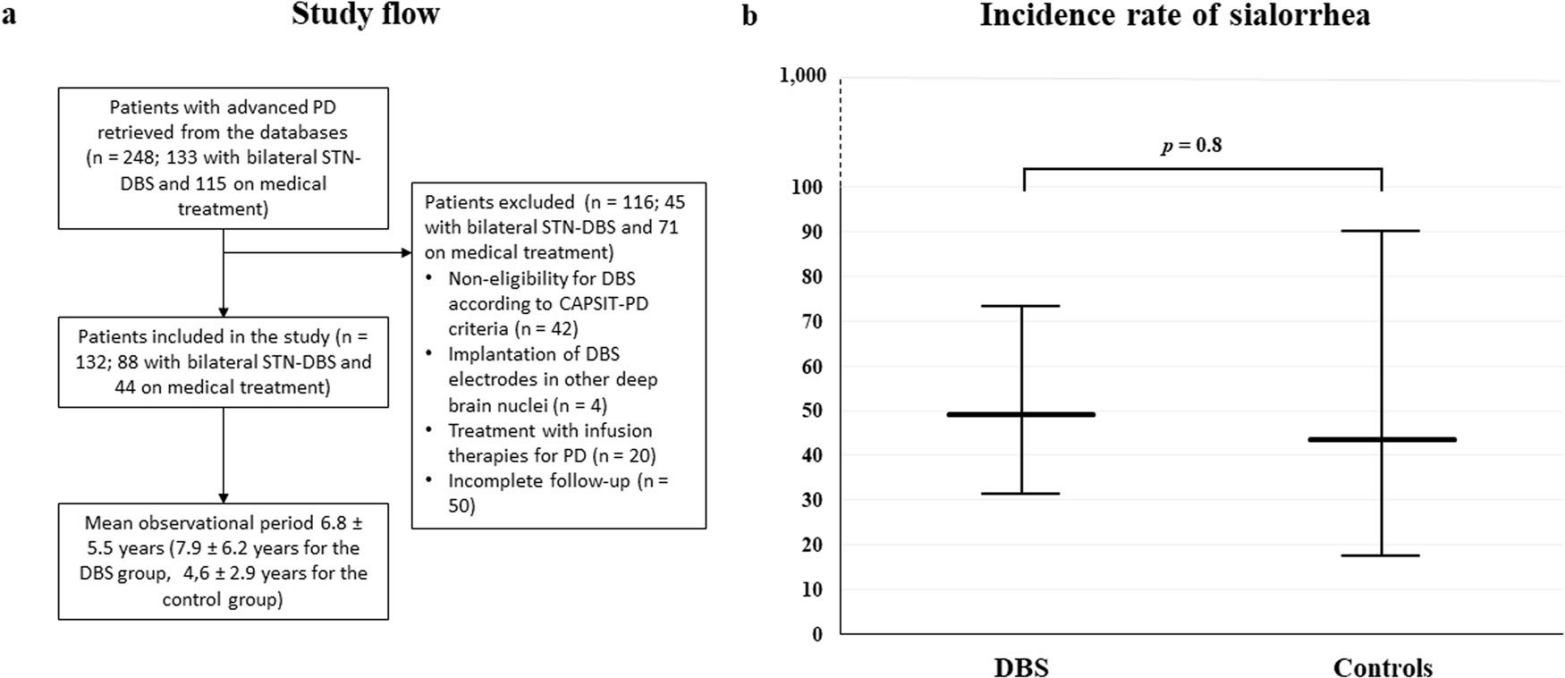
Flowchart of the study and incidence rate of sialorrhea in the two groups of patients. **a** Flowchart of the study. **b** Incidence rate of sialorrhea in the groups of STN-DBS and medically managed patients. The incidence rate of sialorrhea was compared between the two groups using the χ^2^ test.

During follow-up, 31 patients developed new-onset sialorrhea, 24 patients with DBS and 7 patients medically treated. The incidence rate of sialorrhea during the observational period did not differ between the two groups of patients: 49.2 [95% confidence interval (CI) 31.5–73.2] per 1000 person-years of observation in the DBS group and 43.7 (95% CI 17.5–90.2) per 1,000 person-years of observation in the control group (Fig. 1b).

Baseline variables that significantly differed in univariate analysis between patients with and without sialorrhea are shown in Supplementary Table 2. In the final Cox regression model, male sex [hazard ratio (HR) 1.6, 95% CI 1.1–2.3, *p* = 0.006], Hoehn and Yahr stage (HR 2.6, 95% CI 1.3–5.3, *p* = 0.006), and dysphagia (HR 3.5, 95% CI 1.6–7.8, *p* = 0.002) were independent risk factors for sialorrhea (Table 1). In the final regression model, STN-DBS did not significantly increase the risk of developing sialorrhea (HR 1.4, 95% CI 0.7–2.8, *p* = 0.3).

**Table 1:** Baseline risk factors for sialorrhea.

Variable	HR^a^	95% CI (lower-upper)	*P* value
Male sex	1.6	1.1–2.3	**0.006**
Hoehn and Yahr stage	2.6	1.3–5.3	**0.006**
Dysphagia	3.5	1.6–7.8	**0.002**
Disease duration	0.99	0.95–1.05	0.9
UPDRS III off-med	1.0	0.97–1.03	0.9
UPDRS III on-med	1.02	0.99–1.04	0.2
STN-DBS	1.4	0.7–2.8	0.3

*HR* hazard ratio, *CI* confidence interval, *med* medication.

^a^Adjusted for variables in the final Cox regression model: male sex, Hoehn and Yahr stage, dysphagia.

Comparing DBS patients with and without new-onset sialorrhea during the follow-up, no difference was found for stimulation parameters (Supplementary Table 3).

In this retrospective case-control study, focused on the question if DBS-STN may increase the risk of developing sialorrhea in PD patients, the main finding is that the incidence rate of sialorrhea did not significantly differ between the groups of patients with and without DBS in the long-term follow up. To the best of our knowledge, this data has not been previously evaluated in the literature, whereas increased drooling has been reported as an adverse effect in cohorts of stimulated patients^11,12^. Indeed, in our study, the prevalence of sialorrhea at baseline was similar in the two groups of patients (19.3% in the DBS group and 11.4% in the control group). This value is in the lower range reported in PD literature (from 10% to 81%)^7^. This is probably due to selection criteria: in order to avoid selection biases, we included all patients fulfilling the eligibility criteria for DBS^13^, which means patients with advanced disease but without severe axial symptoms and cognitive impairment, which are strongly associated with sialorrhea^6,14^.

In our cohort, the risk factors for sialorrhea were male sex, Hoehn and Yahr stage, and dysphagia. All these variables are known risk factors of sialorrhea in general PD population, as previously described^14–16^. Male sex is a risk factor for PD^17^, and it has been associated with higher severity of PD motor and non-motor symptoms, as sialorrhea^15,16^. Higher scores of Hoehn and Yahr scale represent a more severe disease, with disabling motor symptoms, which are often associated with oro-buccal symptoms as dysphagia, dysarthria and sialorrhea^14^. Dysphagia is a widely recognized causative factor of sialorrhea^18,19^. Interestingly, in our cohort, we have not found an increased or reduced risk deriving from STN-DBS in the long-term follow-up. Moreover, no stimulation parameter was specifically associated with sialorrhea development. All these findings support the hypothesis that sialorrhea is a consequence of the underlying neurodegenerative disease, regardless of DBS.

The main limitations of this study are the retrospective design and the lack of objective measures of sialorrhea. Another limitation is the use in this cohort of anticholinergics and tricyclic antidepressants, which could have an impact on sialorrhea, although these drugs did not significantly differ between the two groups of DBS and medically treated patients.

In conclusion, the present study shows that STN-DBS does not increase the risk of developing sialorrhea, which has a comparable incidence rate in DBS and medically treated patients with advanced PD. This is relevant information that expands our knowledge about the impact of DBS on non-motor symptoms. Indeed, recent evidence suggest the use of DBS in patients with early-stage PD^20^; these are significant results to support the safety of neuromodulation about a disabling symptom such as sialorrhea.

## Methods

This retrospective case-control study was carried out on consecutive PD patients who underwent bilateral STN-DBS from 1993 to 2020 and followed up at the Fondazione Policlinico Universitario A. Gemelli IRCCS in Rome (Italy). At the time of surgery, all patients fulfilled the criteria of idiopathic PD, according to the UK Brain Bank criteria^21^ and the international inclusion and exclusion criteria for DBS^13^. Patients with previous neurosurgical interventions for PD or implantation of DBS electrodes in other deep brain nuclei were excluded from the study. A control group of medically managed patients with advanced PD, followed up at our Centre, was extracted from our databases^22,23^. The patients of the control group were age- and sex-matched with DBS patients with 1:2 ratio. At baseline, each patient included in the control group fulfilled the eligibility criteria for DBS but did not undergo surgery because of refusal by the patient itself or the absence of caregiver. The baseline was defined as the time of surgery for the DBS group and a comparable time-point, matched for disease duration, for the control group. Patients on treatment with infusion therapies for PD or with a follow-up less than six months from the baseline were excluded.

The main outcome of the study was to compare the incidence rate of sialorrhea after the baseline between the groups of STN-DBS and medically managed patients. For each patient, the development of sialorrhea was defined as a score ≥ 2 on item 6 of the Unified Parkinson’s Disease Rating Scale (UPDRS)^24^. The development of sialorrhea was evaluated at each outpatient visit after the baseline (at least twice a year) until the last follow-up available for each patient.

Secondary outcomes included the evaluation of risk factors for sialorrhea and the assessment of post-operative stimulation parameters potentially associated with the development of sialorrhea during the follow-up. To assess the risk factors of sialorrhea, we evaluated at baseline: a) demographic data: sex, age, age at PD onset, disease duration; b) motor scores: Hoehn and Yahr stage^25^, score of part III of the UPDRS in off- and on-medication conditions, axial subscore of part III of the UPDRS (items 27–31) in off- and on-medication conditions; c) medications: mean levodopa equivalent daily dose (LEDD)^26^, therapy with anticholinergics, amantadine, antidepressants, antipsychotics, clozapine; d) stimulation: treatment with STN-DBS; e) motor and non-motor symptoms: presence or absence of dysphagia (score ≥ 2 of item 7 of the UDPRS), presence or absence of speech impairment (score ≥ 2 of item 5 of the UDPRS), psychosis (score ≥ 2 of the item 2 of the UDPRS), orthostatic hypotension (defined as fall in systolic blood pressure of at least 20 mm Hg and diastolic blood pressure of at least 10 mm Hg within 3 min of standing). To assess post-operative stimulation parameters potentially associated with the development of sialorrhea, at the time of development of sialorrhea or at the last follow-up visit for patients without sialorrhea, we evaluated the following variables in the group of DBS patients: stimulation current intensity, pulse width, frequency, setting (single monopolar, double monopolar, bipolar).

The study was approved by the local ethics committee.

### Statistical analysis

Excluding from the analysis of the patients with sialorrhea at baseline, the incidence rate of sialorrhea of the two groups was calculated by dividing the number of new cases by the total number of person-years at risk during follow-up and compared using the χ^2^ test.

To assess the risk factors for sialorrhea, between-group (patients with and without sialorrhea) differences in baseline demographic and clinical variables were analyzed using the Mann-Whitney test for continuous variables and the χ^2^ test or Fisher’s exact test for categorical variables, as appropriate. Variables with *p-*value < 0.05 on univariate analysis were incorporated into a multivariate Cox regression model using a stepwise selection process, building the final regression model with the significant variables.

The between-group (patients with and without sialorrhea) post-operative differences in stimulation parameters were analyzed using the Mann-Whitney test for continuous variables and the χ^2^ test or Fishers’ exact test for categorical variables.

Continuous variables are presented as mean ± standard deviation (SD). All statistical computations were two-tailed, and a *p-*value < 0.05 was considered significant. The statistical analyses were performed using the XLSTAT software, version 2021.3.1 (Addinsoft, Inc., Brooklyn, NY, USA).

## Supplementary information

Supplementary information online

## Data Availability

Anonymized data of this study will be available from the corresponding author on reasonable request from any qualified researcher, following the EU General Data Protection Regulation.
