# Supplementary material for: STN-DBS does not increase the risk of sialorrhea in patients with advanced Parkinson’s disease
Source: NPJ Parkinsons Dis. 2022 Jun 29;8:85. doi: 10.1038/s41531-022-00348-1 (PMC9243012; doi:10.1038/s41531-022-00348-1)
Supplement: Supplementary file 1 — Supplementary information online [file 41531_2022_348_MOESM1_ESM.pdf]

Supplementary information online

Supplementary Table 1. Baseline demographic and clinical data of the two groups of patients (DBS and controls).

	DBS (n=88)	Controls (n=44)	*p* value
**Demographic features**			
Male, n (%)	50 (56.8%)	28 (63.6%)	0.5
Age at baseline (yrs)	56.4 ± 8.4	59.0 ± 7.8	0.1
Age at disease onset (yrs)	43.9 ± 8.4	46.0 ± 7.5	0.1
Disease duration (yrs)	12.5 ± 5.4	12.0 ± 5.0	0.9
**Motor scores**			
Hoehn and Yahr stage	3.0 ± 0.9	2.7 ± 0.6	0.08
UPDRS III off-med	44.6 ± 16.8	35.5 ± 7.8	0.06
UPDRS III on-med	22.4 ± 10.8	21.6 ± 12.8	0.3
Axial subscore UPDRS III off-med	9.8 ± 4.7	7.5 ± 2.9	0.2
Axial subscore UPDRS III on-med	4.7 ± 3.3	3.5 ± 2.9	**0.03**
**Medications**			
LEDD, mg	1015.0 ± 576.2	862.5 ± 338.2	0.2
Anticholinergics, n (%)	20 (22.7%)	12 (27.3%)	0.6
Amantadine, n (%)	33 (37.5%)	15 (34.1%)	0.7
Antidepressants, n (%)	25 (28.4%)	4 (9.1%)	**0.01**
Tricyclic antidepressants, n (%)	3 (3.4%)	0 (0%)	0.6
Antipsychotics, n (%)	22 (25%)	10 (22.7%)	0.8
Clozapine, n (%)	11 (12.5%)	4 (9.1%)	0.6
**Motor and non-motor symptoms**			
Sialorrhea, n (%)	17 (19.3%)	5 (11.4%)	0.2
Dysphagia, n (%)	8 (9.1%)	3 (6.8%)	0.7
Speech impairment, n (%)	31 (35.2%)	14 (31.8%)	0.7
Psychosis, n (%)	37 (42.0%)	13 (29.5%)	0.2
Orthostatic hypotension, n (%)	2 (2.3%)	0 (0%)	0.6

Abbreviations: yrs = years; UPDRS = Unified Parkinson's Disease Rating Scale; med = medications; LEDD = levodopa equivalent daily dose; mg = milligrams.

Group comparisons were assessed using the Mann-Whitney test for continuous variables and the χ^2 test or Fisher's exact test for categorical variables.

Supplementary Table 2. Baseline demographic and clinical variables in patients with and without sialorrhea.

	**Patients with sialorrhea (n=53)**	**Patients without sialorrhea (n=79)**	***p* value**
**Demographic features**			
Male, n (%)	38 (71.7%)	40 (50.7%)	**0.02**
Age at baseline (yrs)	56.5 ± 9.8	57.0 ± 7.1	0.8
Age at disease onset (yrs)	42.8 ± 8.9	46.0 ±7.4	0.05
Disease duration (yrs)	14.0 ± 5.4	12.0 ± 4.7	**0.01**
**Motor scores**			
Hoehn and Yahr stage	3.2 ± 0.8	3.0 ± 0.7	**< 0.001**
UPDRS III off-med	47.0 ±18.4	39.0 ±12.3	**0.0047**
UPDRS III on-med	25.5 ± 13.3	20.0 ± 9.3	**0.007**
Axial subscore UPDRS III off-med	7.4 ± 5.2	9.0 ± 3.9	0.4
Axial subscore UPDRS III on-med	5.1 ± 7.3	4.0 ± 2.6	0.07
**Medications**			
LEDD, mg	965.3 ± 477.3	952.0 ± 523.6	0.6
Anticholinergics, n (%)	12 (22.6%)	20 (25.3%)	0.7
Amantadine, n (%)	18 (34.0%)	30 (38%)	0.6
Antidepressants, n (%)	15 (28.3%)	14 (17.7%)	0.2
Tricyclic antidepressants, n (%)	3 (5.7%)	0 (0%)	0.07
Antipsychotics, n (%)	17 (32.1%)	15 (19.0%)	0.09
Clozapine, n (%)	9 (17.0%)	6 (7.6%)	0.1
**Stimulation**			
STN-DBS patients, n (%)	41 (77.3%)	47 (59.5%)	**0.03**
**Motor and non-motor symptoms**			
Dysphagia, n (%)	9 (17.0%)	2 (2.5%)	**0.003**
Speech impairment, n (%)	23 (43.4%)	22 (27.8%)	0.06
Psychosis, n (%)	24 (45.3%)	26 (32.9%)	0.2
Orthostatic hypotension, n (%)	2 (3.8%)	0 (0%)	0.2

Abbreviations: yrs = years; UPDRS = Unified Parkinson's Disease Rating Scale; med = medications; LEDD = levodopa equivalent daily dose; mg = milligrams; STN-DBS = deep brain stimulation of the subthalamic nucleus.

Group comparisons were assessed using the Mann-Whitney test for continuous variables and the χ^2 test or Fisher's exact test for categorical variables.

Supplementary Table 3. Post-operative stimulation parameters in patients with and without new onset sialorrhea during the follow-up.

**Stimulation parameters**	**Patients with sialorrhea (n=24)**	**Patients without sialorrhea (n=47)**	***p* value**
Current intensity right STN, mA	3.7 ± 1,2	3.9 ± 2.2	0.9
Current intensity left STN, mA	3.5 ± 1,3	4.0 ± 2.1	0.4
Pulse width right STN, μs	61.2 ± 6,1	59.6 ± 6.9	0.7
Pulse width left STN, μs	62.5 ± 8.5	59.6 ± 7.0	0.4
Frequency right STN, Hz	144.0 ± 43.9	130.7 ± 47.8	0.3
Frequency left STN, Hz	143.5 ± 45.2	132.2 ± 48.6	0.3
Single monopolar stimulation right STN, n (%)	14 (58.3%)	17 (36.2%)	0.07
Double monopolar stimulation right STN, n (%)	8 (33.3%)	23 (48.9%)	0.2
Bipolar stimulation right STN, n (%)	2 (8.3%)	7 (14.9%)	0.4
Single monopolar stimulation left STN, n (%)	14 (58.3%)	17 (36.2%)	0.07
Double monopolar stimulation left STN, n (%)	8 (33.3%)	19 (40.4%)	0.4
Bipolar stimulation left STN, n (%)	2 (8.3%)	11 (23.4%)	0.1

Abbreviations: LEDD = levodopa equivalent daily dose; mg = milligrams; mA = milliAmpere; μs = microseconds; Hz = Hertz; STN = subthalamic nucleus.

Group comparisons were assessed using the Mann-Whitney test for continuous variables and the χ^2 test or Fisher's exact test for categorical variables.
